# An integrated dataset on organisational retention attributes and commitment of selected ICT and accounting firms

**DOI:** 10.1016/j.dib.2018.04.140

**Published:** 2018-05-05

**Authors:** Odunayo Salau, Adewale Osibanjo, Anthonia Adeniji, Ebeguki Igbinoba

**Affiliations:** Covenant University, Nigeria

**Keywords:** Retention, Commitment, Reward, Satisfaction, Performance

## Abstract

The article presented an integrated data on organisational retention strategies and commitment of selected ICT and Accounting firms in Nigeria. The study adopted a quantitative approach with a survey research design to establish the major determinants of employee retention strategies. The population of this study included staff and management of the selected firms. Data was analysed with the use of structural equation modelling and the field data set is made widely accessible to enable critical or a more comprehensive investigation. The findings identified critical attraction factors for the retention of sampled firms. It was recommended that ICT firms will need to adopt consistent range of strategies to attract and retain people with the right ICT skills, in the right place and at the right time.

## 1 Introduction

Retention of high performing employees is important and is an essential component for success in an increasingly competitive and demanding environment. Today, organizations are becoming more concerned with employee retention but despite their efforts, employees still leave and this becomes worrisome. Hence, the importance of retaining and maintaining committed employees is especially critical for ICT and Accounting firms in Nigeria.

**Specification Table**

**Table:** 

**Subject area**	Business, Management
**More Specific Subject Area:**	Organizational Behaviour and HRM
**Type of Data**	Primary data
**How Data was Acquired**	Through questionnaire
**Data format**	Raw, analyzed, Inferential statistical data
**Experimental Factors**	Population consisted of selected ICT and Accounting firms in Nigeria. The researcher-made questionnaire which contained data on retention strategies and employee commitment.
**Experimental features**	Retention of high performing employees is important and is an essential component for success in an increasingly competitive environment.
**Data Source Location**	Lagos, Nigeria
**Data Accessibility**	Data is included in this article

**Value of data**

• The data can be used by managers to properly make decisions that in the long-run would lead to goal attainment in the organization.
• The data can be used to enlighten managers on the importance of retention attributes and how it can be beneficial to the overall wellbeing of the organization.
• The data provides ample knowledge on how different organisational retention attributes can interact effectively by building healthy relationship and sustaining greater commitment.
• Generally, data acquired from this study would be significant for organizational goal achievement, proper building of corporate image which would in turn lead to organizational success.
• The data described in this article is made widely accessible to facilitate critical or extended analysis.

## 2 Data

**Fig. 1:**
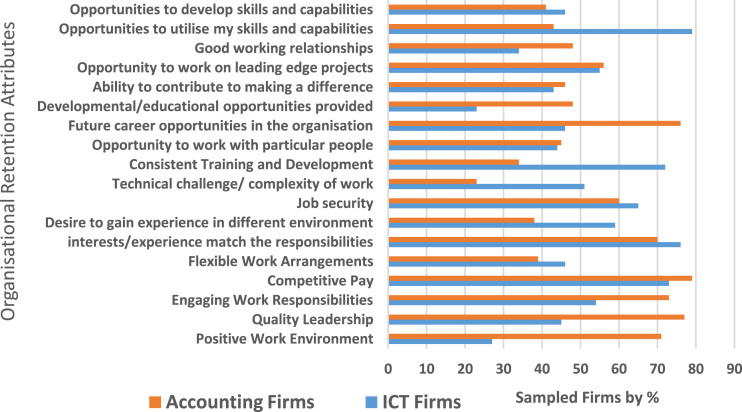
Retention attributes of the sampled firms.

**Fig. 2:**
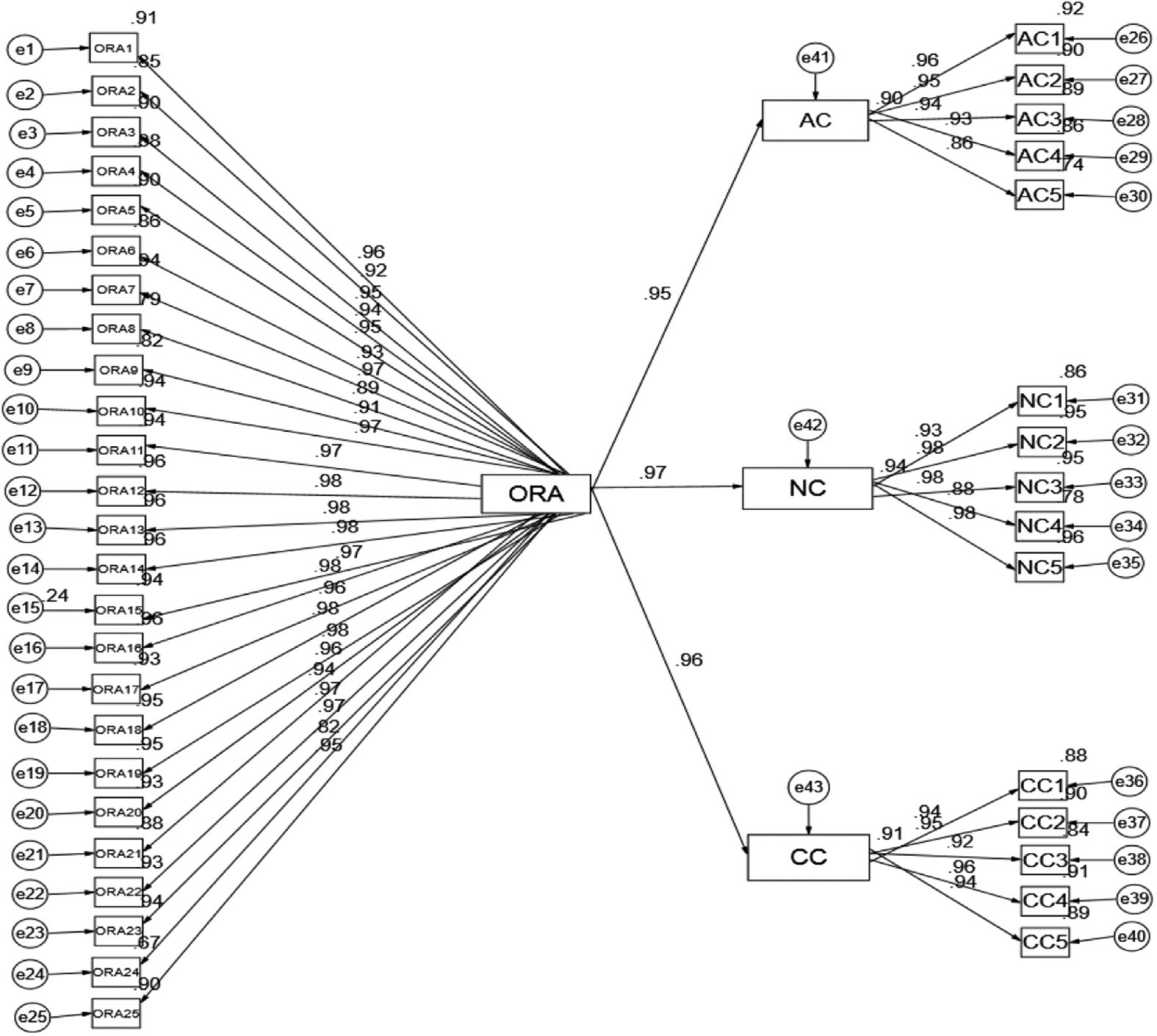
Regression weights of the variables.

The study is quantitative in nature and data were retrieved from staff and management of the sampled firms. The decision to elicit information from the employees and the management group was based on the fact that while employees were often in the best position to describe their real employment relationships and knowledge of retention practices as presented in Fig. 1. The study also adopted the approach recommended by Anderson and Gerbing (1998) to evaluate: (1) measurement model and (2) structural model. To demonstrate the measurement model, we used Confirmatory Factor Analysis (CFA) and the three conditions for CFA loadings indicate firstly, that all scale and measurement items are significant when it exceeds the minimum value criterion of 0.70; second, each construct composite reliability exceeds 0.80 and thirdly, each construct average variance extracted estimate (AVE) exceeds 0.50, as presented in Table 1 and Fig. 2 respectively.

**Table 1:** Demonstrated convergent reliability: the researchers used CFA to assess composite reliability and the average variance extracted (AVE) of the specific constructs.

**Measurement**		**Loading**	**Indicator Reliability**	**Error Variance**	**Sum of Variance**	**Compose Reliability**	**Ave. Variance Estimated**
		**> 0.7**		**< 0.5**		**> 0.8**	**> 0.5**
Organisational Retention Strategies							
ORA1		0.956	0.9139	0.0861	2.3885	0.9958	0.9045
ORA2		0.922	0.8501	0.1499			
ORA3		0.948	0.8987	0.1013			
ORA4		0.937	0.8780	0.1220			
ORA5		0.949	0.9006	0.0994			
ORA6		0.927	0.8593	0.1407			
ORA7		0.969	0.9390	0.0610			
ORA8		0.887	0.7868	0.2132			
ORA9		0.908	0.8245	0.1755			
ORA10		0.970	0.9409	0.0591			
ORA11		0.972	0.9448	0.0552			
ORA12		0.981	0.9624	0.0376			
ORA13		0.980	0.9604	0.0396			
ORA14		0.980	0.9604	0.0396			
ORA15		0.971	0.9428	0.0572			
ORA16		0.980	0.9604	0.0396			
ORA17		0.964	0.9293	0.0707			
ORA18		0.975	0.9506	0.0494			
ORA19		0.977	0.9545	0.0455			
ORA20		0.962	0.9254	0.0746			
ORA21		0.940	0.8836	0.1164			
ORA22		0.966	0.9332	0.0668			
ORA23		0.968	0.9370	0.0630			
ORA24		0.820	0.6724	0.3276			
ORA25		0.950	0.9025	0.0975			
	**Affective Commitment (AC)**						
AC1		0.958	0.9178	0.0822	0.6916	0.9689	0.8617
AC2		0.949	0.9006	0.0994			
AC3		0.944	0.8911	0.1089			
AC4		0.927	0.8593	0.1407			
AC5		0.860	0.7396	0.2604			
	**Normative Commitment (NC)**						
NC1		0.925	0.8556	0.1444	0.4993	0.9783	0.9001
NC2		0.977	0.9545	0.0455			
NC3		0.976	0.9526	0.0474			
NC4		0.884	0.7815	0.2185			
NC5		0.978	0.9565	0.0435			
	**Continuance Commitment (CC)**						
CC1		0.937	0.8780	0.1220	0.5736	0.9747	0.8853
CC2		0.951	0.9044	0.0956			
CC3		0.917	0.8409	0.1591			
CC4		0.956	0.9139	0.0861			
CC5		0.943	0.8892	0.1108			

The results of CFA analysis suggest that the factor loadings for all major variables range between 0.820 and 0.981. The three conditions used to assess convergent validity as suggested and recommended by Fornell and Larcker [4] and Bagozzi and Yi (1988) were met. Details of the results are available in Table 2, which exhibit that the coefficient correlation is highly correlated and are all significant.

**Table 2:** Discriminant validity.

	ORA	AC	NC	CC
ORA	**0.9510**	0.3914**	0.2682**	0.1676**
AC		**0.9283**	0.3341**	0.1193**
NC			**0.9488**	0.6404**
CC				**0.9409**

The diagonal values represent the square root of the average variance extracted (AVE) of the specific construct.

Construct legend: ORA_ Organisational Retention Attributes; AC_ Affective Commitment; NC_ Normative Commitment; CC_ Continuance Commitment.

⁎⁎ Correlation is significant at the 0.01 level (2-tailed)

Based on the results of the test, it has been proven that the data are good in terms of convergent validity, construct reliability, and discriminant validity. Having run the test, the SEM was obtained, and results of fit indices is shown in Table 3.

**Table 3:** The model fit summary showing the goodness of fitness.

Goodness of fit	SEMs Value	Recommendation Values	Remarks
Chi­Square/Degree of Freedom (CMIN/DF)	2.629	≤ 3.00	Acceptable fit
Normed Fit Index (NFI)	0.922	≥ 0.90	Good fit
Comparative Fit Index ( CFI)	0.984	≥ 0.90	Very Good fit
Incremental Fit Index (IFI)	0.934	≥ 0.90	Good fit
Root Mean Squared Error of Approximation (RMSEA)	0.061	≤ 0.08	Good fit
Goodness of Fit (GFI)	0.933	≥ 0.90	Good fit

Results in Table 3 dictate that the value of *χ*2 is within the acceptable range of 1 and 3 as suggested by Brown and Cudeck (1993) and Hu Bentler (1999). On top of that, the incremental fit, NFI, TLI, CFI, and GFI were above 0.90 (Bentler and Bonnet, 1980; Bagozzi and Yi, 1998). Meanwhile, results for standardised regression weights for each variable are stated in Table 4.

**Table 4:** Standardized regression weights.

**Dependent**		**Independent**	**Estimate**	**S.E.**	**C.R.**	***P***	**Label**
AC	<---	ORA	0.950	0.010	72.670	***	Sig
NC	<---	ORA	0.971	0.010	97.069	***	Sig
CC	<---	ORA	0.955	0.011	77.427	***	Sig.

All the basic assumptions were acceptable and prove that the data met the conditions of basic assumption in regression analysis.

## 3 Experimental design, materials and methods

Of the 418 copies of questionnaire distributed, 376 responses were received, resulting in a response rate of 89.9%. Members of selected five (5) ICT and five (5) Accounting firms were represented in this study. Data were gathered from directors, managers, assistant managers, scientists, field agents, and other categories of employees across the various ICT and Accounting firms with the aid of a researcher- made questionnaire based on the works of [1], [2], [3], [4], [5], [6]. The demographic data presented information based on gender, age, education and experience as well as questions related to organisational retention attributes and staff commitment. There was a meaningful relationship between organisational retention attributes and the commitment of staff in the selected firms. The collected data were coded and analysed using SPSS version 22. Data was analysed applying descriptive and inferential statistical tests. Importantly, the study participants were selected based on the following inclusion criteria:

**Inclusion criteria:**

• Participants were employees of the sampled ICT and Accounting firms.
• Participants were literate, able to read and write English.
• Participants signed the consent form provided and have worked with the firm for a minimum period of 3 years.
• Participants were accessible as at the time of the survey and interviews.

As regards retention, items used included: the main reasons for participants agreeing to work within the firm; whether a detailed job description was given on appointment with the organization, and if the job description tallied with the real job done; the existence of a clearly specified daily job description; retention strategies adopted; relevance of regularly conducted trainings/workshops; and the existence of the desire to change jobs. The section on commitment was adapted from a previously validated questionnaire – the Organizational Commitment Questionnaire, OCQ.

## 4 Ethical considerations

The researchers ensured that respondents were well informed about the background and the purpose of this research and they were kept abreast with the participation process. Respondents were offered the opportunity to stay anonymous and their responses were treated confidentially.
