# Supplementary material for: An integrated dataset on organisational retention attributes and commitment of selected ICT and accounting firms
Source: Data Brief. 2018 May 5;18:1930–6. doi: 10.1016/j.dib.2018.04.140 (PMC5998706; doi:10.1016/j.dib.2018.04.140)
Supplement: Supplementary file 1 — Supplementary material [file mmc1.docx]

**DECLARATION OF INTEREST FORM**

**AN INTEGRATED DATASET** [**ON ORGANISATIONAL RETENTION ATTRIBUTES AND COMMITMENT OF SELECTED ICT AND ACCOUNTING FIRMS**](https://www.sciencedirect.com/science/article/pii/S2352340917302949)

Odunayo **SALAU**; Covenant University

[odunayo.salau@covenantuniversity.edu.ng](mailto:odunayo.salau@covenantuniversity.edu.ng)

Adewale OSIBANJO; Covenant University

adewale.osibanjo@covenantuniversity.edu.ng

Anthonia ADENIJI; Covenant University

anthonia.adeniji@covenantuniversity.edu.ng

Ebeguki IGBINOBA; Covenant University

ebe.igbinoba@covenantuniversity.edu.ng

We, the Authors of paper entitled above certify that we have seen and approved the final version of the manuscript being submitted. This is an original work and has not received prior publication and is not under consideration for publication elsewhere. It is also important to state that there is no financial/personal interest or belief that could affect our objectivity and to prevent ambiguity, we humbly want to state explicitly that there is no conflicts of interest as regards the review and publication of this paper.

Thank you.

SALAU Odunayo

*Signed*
